# Contribution of Topical Agents such as Hyaluronic Acid and Silver Sulfadiazine to Wound Healing and Management of Bacterial Biofilm

**DOI:** 10.3390/medicina58060835

**Published:** 2022-06-20

**Authors:** Francesco De Francesco, Michele Riccio, Shiro Jimi

**Affiliations:** 1 Department of Reconstructive Surgery and Hand Surgery, University Hospital (AOU Ospedali Riuniti di Ancona), Via Conca 71, 60126 Ancona, Italy; michele.riccio@ospedaliriuniti.marche.it; 2 Department of Plastic and Reconstructive Surgery, Faculty of Medicine, Fukuoka University, Fukuoka 814-0180, Japan; sjimi@fukuoka-u.ac.jp

**Keywords:** chronic wounds, wound infections, silver, sulfadiazine, hyaluronic acid, antisepsis

## Abstract

*Background and Objectives:* Wound healing is commonly associated with critical bacterial colonization or bacterial infection, which induces prolonged inflammation, resulting in delayed re-epithelialization. An appropriate wound dressing requires a humid environment, which also functions as a barrier against bacterial contamination and will accelerate a regenerative response of the wound. Silver sulfadiazine (SSD) is used to prevent wound infection. Hyaluronic acid (HA) is an extracellular matrix component involved in tissue regeneration. This retrospective study was conducted to evaluate the effectiveness of cream and gauze pads based on hyaluronic acid at low molecular weight (200 kDa) and silver sulfadiazine 1% in the wound healing process. In addition, we examined SSD action on biofilms in vitro and on animal wounds, obtaining positive outcomes therefrom. *Materials and Methods:* We selected 80 patients with complicated chronic wounds of different etiologies, including diabetes mellitus (10), post-traumatic ulcers (45), burns (15), and superficial abrasion (10). *Results:* After 8 weeks, ulcer size was decreased in 95 ± 2% of the treated patients; a significant reduction in the inflammatory process was observed from day 14 onwards (*p* < 0.01 vs. baseline), considering improvement of the surrounding skin and reduction of the bacterial load. The SSD treatment decreased bacterial colony proliferation, both in planktonic state and in biofilm, in a dose-dependent manner on the wound but inhibited the development of tissue granulation at the highest dose (800 μg/wound). *Conclusions:* In conclusion, the combined action of SSD and HA is clinically effective in improving wound healing.

## 1. Introduction

The majority of hard-to-heal ulcers present bacterial contamination, which commonly results in delayed healing [1]. Contamination is to be distinguished in saprophytic colonization of the ulcer and infection. If neglected, contamination may progress to local infection and, in immunocompromised individuals, to systemic infection, sepsis and multi-organ dysfunction syndrome [2]. The presence of microbial contamination in chronic and acute wounds is difficult to define due to the presence of a saprophytic biofilm, which may trigger inflammatory activity, and due to abundant and sustained nitric oxide stimulation and free radicals and inflammatory cytokines levels, causing a delay in the healing process. Biofilm control plays an important role in the treatment of chronic wounds [3]. Complex ulcers require careful clinical analysis with a clear identification of local infection indicators. The preparation of the wound bed (Wound Bed Preparation) is fundamental in chronic wound management. The TIME framework, i.e., Tissue, Infection or Inflammation, Moisture imbalance, Epidermal margin [4,5,6], is an appropriate tool to identify four clinical areas to consider in wound bed preparation, which correspond to pathophysiological anomalies requiring correction to facilitate the physiological healing process. Despite proper management of a hard-to-heal ulcer, infection of the ulcer with associated inflammation is a probable cause of delayed healing, notwithstanding the availability of a broad spectrum of antimicrobials for prophylaxis and treatment [7]. For such chronic wounds, the initial management generally involves aggressive surgical debridement, hyperbaric oxygen therapy, and vacuum therapy [2,8], but the use of topical dressings to manage biofilms and prevent inflammation progression requires further investigation.

Microbiological investigations in acute soft tissue infections have revealed that *Staphylococcus aureus* is the specific bacterium in approximately 30% of cutaneous abscesses [9], *Staphylococcus aureus*, *Streptococcus pyogenes*, and *Escherichia coli* are present above all in traumatic injuries or necrotizing soft tissue infections [10], *Pseudomonas aeruginosa*, *Staphylococcus aureus*, *Escherichia coli*, *Klebsiella*, *Enterococcus* and *Candida* are, moreover, a challenging issue for burn injuries [11,12], and *Staphylococcus aureus*, *Staphylococcus epidermidis*, *Streptococcus*, *Pseudomonas aeruginosa*, *Enterococcus* and coliform bacteria [13,14,15] are common in diabetic and chronic leg wounds. In particular, the clinical inference of microorganisms incorporated in the biofilm include moderate and non-specific symptoms, with high resistance and persistence of infections resistant to antimicrobial agents. To avoid such complications, many medical professionals have turned their attention to antimicrobial therapeutic alternatives to prevent microbial contamination, focusing specifically on topical antibiotic dressings, such as iodine-based preparations, cadexomer iodine, polyhexamethyl biguanide (PHMB), hydrogels and honey. However, the overreliance on antibiotics and the widespread resistance to such therapy is becoming an increasing concern for many global health organizations. HA is a glycosaminoglycan and is the main component of the extracellular matrix, with numerous effects including an anti-inflammatory action over the recruitment of inflammatory cells and cytokines, the migration of stem cells [16], the elimination of intracellular reactive oxygen radicals (via CD44 intracellular signaling pathways) [17], but above all, it is responsible for adhesion to ECM components. Clinically, HA diminishes the contracture in wound scars [18] and favors collagen expression. In particular, different HA-based wound dressings are used to increase the efficacy of drugs and growth factors and the release of antimicrobial agents. Studies have indeed shown a greater antimicrobial activity of silver particles in association with hyaluronan [19,20] in the treatment of wounds and burns, which promotes the healing process through cell growth and migration attributable to the presence of HA, but also by preventing microbial contamination due to the silver constituent. Herein discussed is a topical preparation whose main component is hyaluronic acid. Silver sulfadiazine present in the product composition plays a key role in preventing microbial contamination, thus improving hyaluronic acid ability to support wound healing. These characteristics allow only for one to two dressing changes per day, causing less discomfort to the patient and minimum air exposure of the lesion, thus reducing the possibility of wound contamination. The main objective of this retrospective study was to assess the clinical impact of hyaluronic acid (200 kDa) and silver sulfadiazine 1% in the treatment of chronic wounds, in terms of wound size reduction and bacterial load control.

## 2. Materials and Methods

A single-center, observational and retrospective study was conducted from January 2017 to December 2020 in outpatients with chronic wounds complicated by microbial contamination to evaluate the effectiveness of a cream and cream-impregnated gauze pads containing hyaluronic acid (200 kDa) and silver sulfadiazine 1% (Fidia Farmaceutici, Abano Terme, Italy) in the wound healing process. The study was performed in compliance with the Declaration of Helsinki and the Guidelines for Good Clinical Practice. The study protocol was approved by the Marche Regional Ethics Committee (CERM-Italy) (397/2021). All bacterial culture methods were performed in accordance with the relevant guidelines and regulations at Fukuoka University.

### 2.1. Clinical Investigation

Patients aged 18 years or over and affected by contaminated chronic wounds of different etiologies were considered eligible to participate in the study. The main inclusion criteria were the presence of incomplete or total loss of substance with no clinical signs of overt infection but signs of bacterial contamination such as modification in the appearance of the granulation tissue and delayed wound healing. Eighty patients were enrolled, presenting contaminated hard-to-heal wounds of different etiologies, including diabetes mellitus (10), post-traumatic ulcers (45), burns (15), and superficial abrasion (10). The general characteristics of the patients are described in Table 1.

The lesions were treated with cream or cream-impregnated gauze pads based on low molecular weight (200 kDa) hyaluronic acid and silver sulfadiazine 1% (Connettivina Bio Plus, Fidia Farmaceutici SpA, Abano Terme Italy). The treatment type was determined by the treating physician for each patient according to the wound type and the depth and extension of the ulcer. The depth of the ulcers treated with the gauze pads ranged from 0.1 to 1 cm, while the depth of the ulcers treated with the cream ranged from 1 to 1.3 cm. In most patients, the wounds were located on the upper and lower limbs.

All the patients underwent the same therapeutic protocol: wound disinfection, washing with saline solution, and application of hyaluronic acid/silver sulfadiazine cream or gauze pads to the wounds. All ulcers were dressed twice a week at our Clinic, while we performed follow-up visits (imaging, wound characterization, size, edges, presence of exudate, periwound skin evaluation, bacterial swab). The treatment continued until the wound was clean and granulating. No advanced dressing was used. The wounds were diagnosed as completely healed when the normal reepithelization process was complete. Follow-up visits were conducted twice a week until the ulcer was completely healed, according to AHRQ recommendations [21].

#### 2.1.1. Clinical Evaluation of Antibacterial Effects

The wounds were cleansed prior to the collection of the wound swabs. We used the Levine technique [22] to collect samples from the wounds. The swab samples were collected at baseline, after two weeks, and after four weeks to evaluate any reduction/variation in colony-forming units (CFUs).

#### 2.1.2. Assessment of Efficacy

The primary efficacy criterion was the reduction of the wound surface area during the study. Changes in wound size are difficult to assess by wound length and width due to the irregular shape of wounds; hence, we measured the wound surface area in cm^2^ using squared and transparent paper on which the wound area was traced with indelible marker pens (Oxford Health NHS Foundation Trust guidelines).

The secondary efficacy criterion consisted in evaluating changes to the characteristics of the wound in terms of direct rate of complete ulcer healing, appearance of the skin around the wound, and prevention of microbial contamination. Successful wound closure rate was defined as at least 95% epithelization of the wound in relation to the original surface area of during the study. The appearance of the surrounding skin was evaluated by changes according to Harikrishna Periwound Skin (HPs) classification [23]. All parameters were assessed at baseline and at 2, 4, 8 weeks of treatment (Table 2).

#### 2.1.3. Assessment of Tolerability and Safety

Treatment tolerability was assessed based on wound redness, irritation, pruritus, or pain. Adverse events (AE) were recorded from study initiation to study termination. All adverse events are reported as separate events, including signs, symptoms, laboratory findings, or disease and were monitored until complete recovery or conclusive physical examination and assessment.

### 2.2. Experimental Study

#### 2.2.1. Bacteria and Biofilm Chips

A clinically isolated *S. aureus* biofilm former was cultured in Tryptic Soy Broth (Becton, Dickinson and Company Japan, Tokyo, Japan) and selected for analysis. *S. aureus* biofilms formed on plastic chips (1 × 1 cm) after 34 h of incubation at 37 °C were used.

#### 2.2.2. Evaluation of Antibacterial Effects

The effects of reagents on *S. aureus* in planktonic and biofilm cultures were analyzed by methods previously employed [24]. SSD (ALCARE Co. Ltd., Tokyo, Japan) and vancomycin (Sigma Aldrich Japan, Tokyo, Japan) were utilized. Maximum concentrations were 4000 μg/mL for SSD and 200 μg/mL for vancomycin. Minimum inhibition concentration (MIC) for planktonic bacteria, minimum bactericidal concentration (MBC) for planktonic bacteria on tryptic soy ager (TBA), and minimum biofilm eradication concentration (MBEC) for bacteria in biofilms on TBA were analyzed after 24 h of incubation using halving reagent serial solutions.

#### 2.2.3. Effects of SSD on Mice Wounds

The conducted animal experiments received prior approval by the board of the Center for Experimental Animals at Fukuoka University (#1210608). Our established wound infection model [25] was applied. The back skin (2 cm) was roundly excised at the radius in female subjects C57BL/6N (Japan SLC Co. Ltd., Shizuoka, Japan) of 11 weeks of age, and the wounds were covered with a skin adhesive. For bone marrow myelosuppression, 5-fluorouracil (5-FU) at a dose of 2.25 mg/100 μL/2 g body weight (Sigma Aldrich Japan, Tokyo, Japan) was intraperitoneally injected on day 0. An *S. aureus* suspension at a density of 4 × 10^7^ CFU/200 μL was inoculated into the wound space on day 3. On day 4, SSD was injected into the wound space at concentrations of 0, 80, 320, and 800 μg/200 mL/mouse. As a control, SSD injection without bacterial inoculation was performed. Each study group consisted of three animals. On day 6, all animals were euthanized by cervical dislocation, and histological samples were prepared. Thin sections of paraffin blocks including tissue were dyed with hematoxylin–eosin and Gram stains.

### 2.3. Statistical Analysis

Statistically significant differences were calculated by the Student’s *t*-test. The threshold for statistical significance was based on a *p*-value < 0.05. The statistical analysis software (SPSS) was used to conduct all the statistical tests (the two-tailed Student’s *t*-test along with the Kolmogorov–Smirnov test).

## 3. Results

### 3.1. Clinical Study of Ulcers Treated with SSD + HA

Data were collected between January 2017 and December 2020 regarding 80 patients enrolled in a single-center study. On examination of wound types and localization, we observed that 56% of the wounds were described as trauma with loss of substance, 19% as first- or second-degree burns, 12.5% as diabetic ulcers, and 12.5% as superficial abrasions.

A cream formulation was used to treat 55 wounds, and a gauze formulation was applied to 25 wounds.

The most common bacterial species isolated via a swab (according to the Levine technique) was *Staphylococcus aureus*, followed by *S. epidermidis*, *S. lugdunensis*, *E. faecalis*, *K. pneumoniae*, and *P. aeruginosa*.

**Figure 1:**
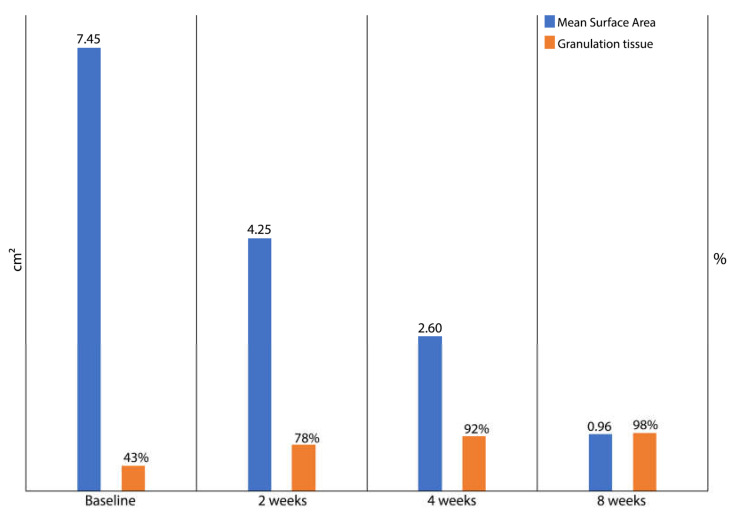
Progression of wound healing during the treatment with an ointment based on hyaluronic acid and silver sulfadiazine, as measured by the mean wound surface area and the overall mean percentage of granulation tissue. The mean wound surface area decrease is inversely proportional to the increase in the percentage of granulation tissue.

At baseline, the mean surface area of the wounds measured 7.45 cm^2^ ± 1.32 cm^2^ (mean ± SD). The mean time between wound occurrence and treatment initiation was 5 days. After 4 weeks of treatment, wound size reduction (primary endpoint) was statistically significant, with a mean percentage of 65% (*p* = 0.003), observing a decrease in wound size from a mean value of 7.45 cm^2^ at baseline to 2.60 cm^2^ (Figure 1). A significant wound healing rate was observed at each evaluation compared to baseline: at 2 weeks, we observed a 43% reduction (100% vs. 57%; 95% confidence interval (CI) = 48%–38%), at 4 weeks, a 65% reduction (100% vs. 35%; 95% CI = 70%–60%), at 8 weeks, an 87% reduction (100% vs. 13%; 95% CI = 92%–82%). Moreover, healing occurred in the majority of patients (80% ± 4%), and the established ulcer size reduction was achieved in 95% ± 2% of the patients after 8 weeks of treatment.

**Figure 2:**
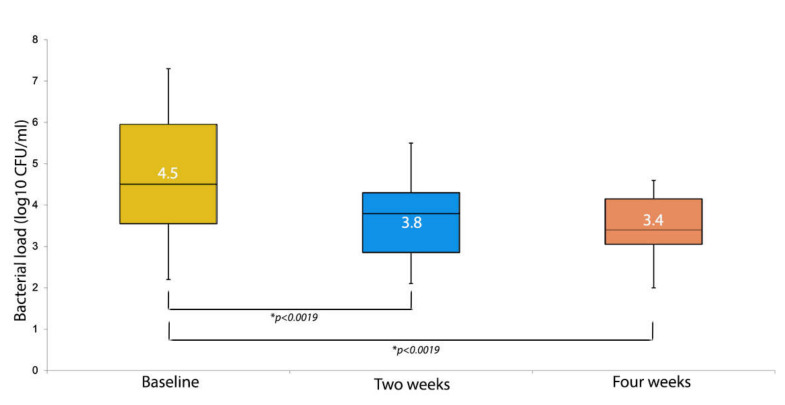
Bacterial load at each visit (log10 CFU/mL). For each box plot, the median values are represented by the line within the box. The box represents 50% of the values (the 25th and 75th centiles), with the bars indicating the highest and lowest values excluding the outliers. * is *p* value.

Regarding the secondary endpoints, at baseline the median bacterial load was 4.5 log10 CFU/mL, and this value decreased to 3.8 log10 CFU/mL after two weeks and to 3.4 log10 CFU/mL after four weeks (Figure 2).

After 4 weeks from the initiation of the hyaluronic acid and silver sulfadiazine therapy, we observed a significant reduction of the bacterial load present in the ulcers that was directly proportional to the increase in granulation tissue and, consequently, to the reduction of the ulcer area. Moreover, the overall mean percentages of granulation tissue after 14 days were 78% compared to 43% at baseline.

**Figure 3:**
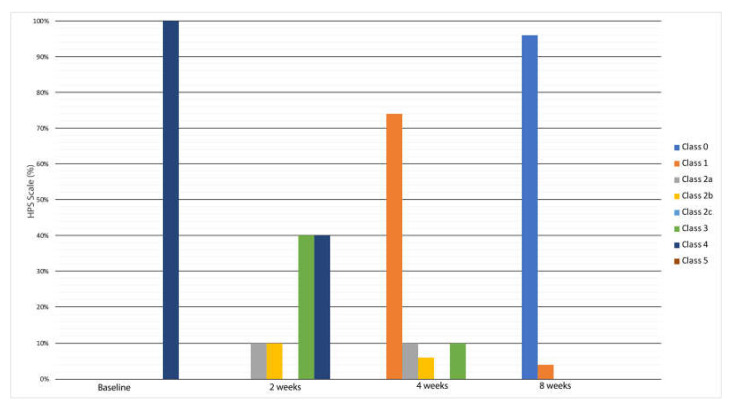
Evaluation of the quality of the wound-surrounding skin during treatment applications by the HPS score. All wounds progressively changed from class 3 to class 1 after 4 weeks.

Moreover, after 4 weeks, absence of inflammation was observed in 74% of the patients, and the decrease in inflammation was significant from day 14 onwards (*p* < 0.01 vs. baseline at day 14 and *p* = 0.001 vs. baseline at 4 weeks) (Figure 3). Management of the periwound skin is crucial in wound healing, as keratinocytes usually migrate from the periwound area. Our data showed that the periwound tissue of the ulcers included in the study (100%) were graded as class 3, i.e., with the presence of inflammation without clinical signs of infection. After 2 weeks of treatment, the periwound tissue showed clinical improvement and was therefore graded as class 2a (20%), in which the presence of exudate with desiccation was highlighted, and then as class 2b (15%), in which the presence of exudate with maceration was highlighted. Following 4 weeks of treatment, the periwound tissue showed a marked improvement, such that 74% of it was normal (class 0).

**Figure 4:**
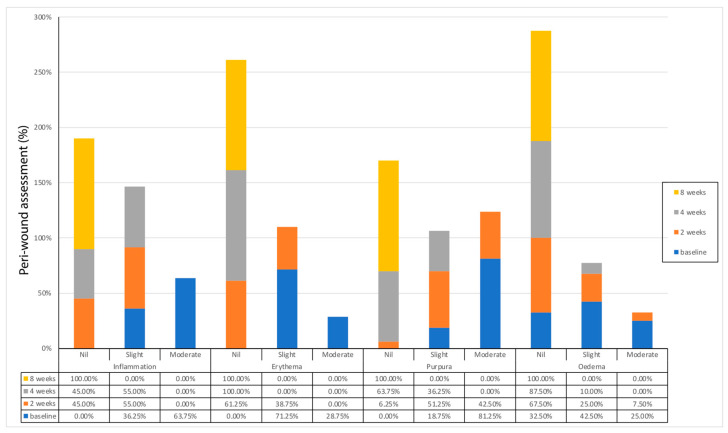
Evaluation of peri-wound inflammation, erythema, purpura, oedema. After 2 weeks, 45% of the treated ulcers showed an improvement in the four parameters analyzed, and at 4 weeks, 74% of the treated ulcers showed further improvement in the four parameters compared to baseline.

A significant change in inflammation, erythema, purpura, and oedema was observed during the treatment compared to baseline (Figure 4).

Pain reduction after 8 weeks was marked in all patients, with a statistically significant reduction (data not shown, *p* < 0.001). In fact, the cream and gauze formulations containing hyaluronic acid and silver sulfadiazine were well tolerated by the patients. According to our results, the majority of the patients were fully satisfied with the formulations. No significant differences were observed in healing time considering either formulation regarding both tolerance and overall patient satisfaction. No AEs or SAEs were reported in relation to the procedures or to the experimental product.

**Figure 5:**
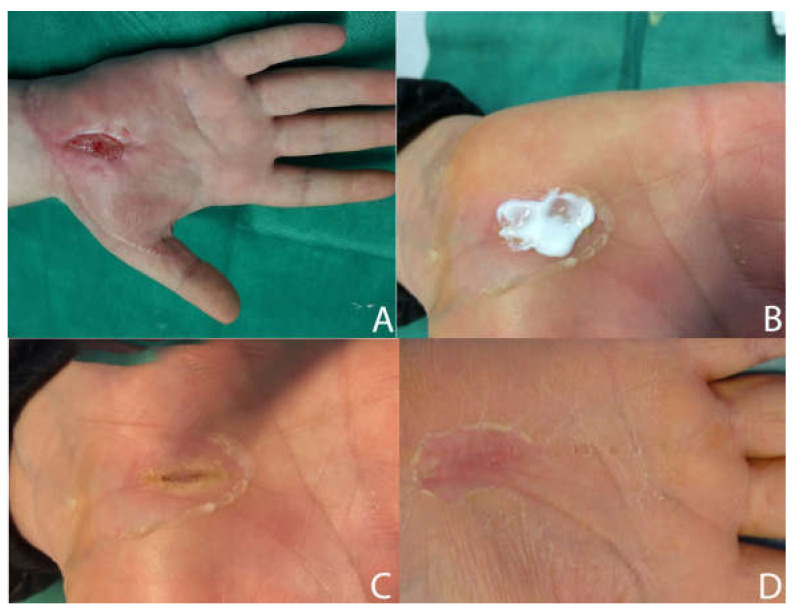
Post-traumatic case. AC, 52 y/o. Presence of periwound inflammation signs and Class 3 periwound tissue according to HPS classification. Appearance of the wound after injury (**A**). Application of the Connettivina Plus ointment (**B**). Wound aspect after 2 weeks (**C**) and final aspect with complete reepithelialization after 8 weeks (**D**).

**Figure 6:**
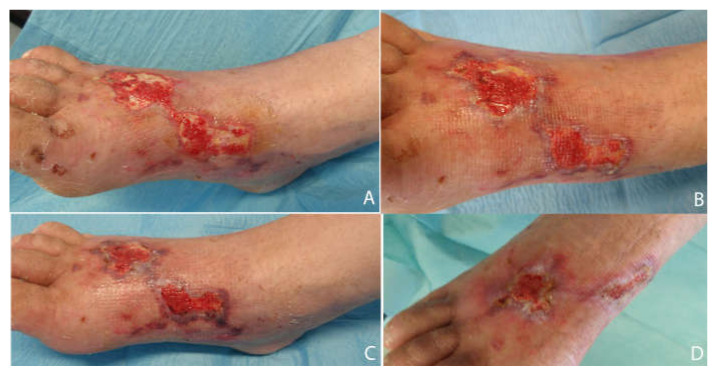
Diabetic case. AA, 58 y/o. Presence of periwound inflammation sign and Class 3 periwound tissue by HPS classification. Appearance of the wound before the treatment (**A**). Wound aspect after 2 weeks (**B**). Wound aspect after 4 weeks (**C**) and final aspect with partial reepithelialization after 8 weeks (**D**).

**Figure 7:**
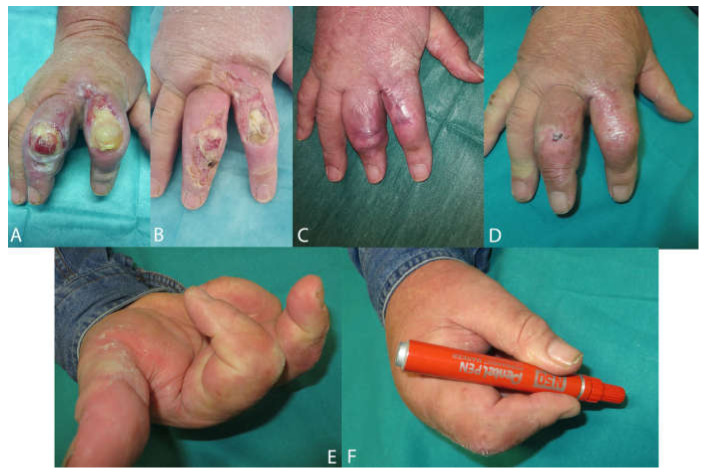
Burn case. MR, 70 y/o. Presence of periwound inflammation sign and Class 3 periwound tissue by HPS classification. Appearance of the wound before the treatment (**A**). Wound aspect after 2 weeks (**B**). Wound aspect after 4 weeks (**C**) and final aspect with complete reepithelialization after 8 weeks (**D**). Good motor function of the hand (**E**,**F**).

Figure 5, Figure 6 and Figure 7 show examples of clinical cases.

### 3.2. Experimental Study

In the light of these findings, SSD was hypothesized to be effective in promoting the healing of ulcers in patients with bacterial infections. We proceeded to further assessments using: (1) a wound healing mouse model and (2) the evaluation of SSD bactericidal effects compared to a common antibacterial agent.

#### 3.2.1. Bactericidal Effects of Vancomycin and SSD

MIC and MBC indicate the concentrations of a reagent causing bacteriostasis and bacterial killing of bacteria in the planktonic state, respectively. For vancomycin, we observed a 32-fold increase of the MBC/MIC ratio for biofilm compared to planktonic bacteria (Table 3). The MBC/MIC and MBEC/MIC showed similar results for vancomycin. For SSD, MBC/MIC was 4, and MBEC/MIC was 8; the values were more than four times lower for SSD than for vancomycin.

#### 3.2.2. SSD Effects on Wound Infection in Mice

**Figure 8:**
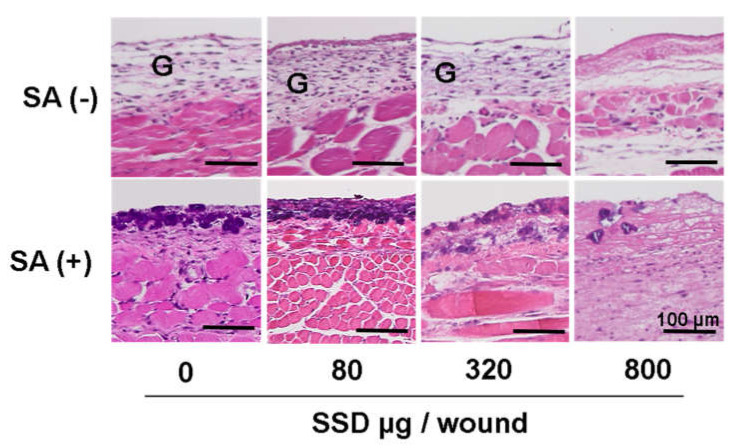
The effects of SSD on *S. aureus*-infected wounds in mice. *S. aureus* (SA) was inoculated 3 days after back skin excision, and SSD in different doses was applied onto the wound surface on day 4. Wound tissues stained by Gram + HE staining are shown on day 6. G: granulation tissue; bars = 100 μm.

After inoculating *S. aureus*, bacteria formed biofilm-associated colonies on the wound surface. Representative pictures of each group are displayed in Figure 8. A higher bacterial density accumulated when using the SSD dose of 80 μg/wound. The bacterial density decreased when SSD was used at a dose of more than 320 μg/wound; however, granulation tissue did not form well at the dose of 800 μg SSD/wound.

## 4. Discussion

The control of biofilm is one of the main problems in chronic wound management. The use of antimicrobial dressings for infection management is worthy of further investigation. The concept of bacterial contamination, colonization, and infection in wound care is now universally acknowledged, and the correlation between biofilm and chronicity of ulcers is strictly related to an incorrect therapeutic approach [26]. Most chronic wounds are defined by a polymicrobial aerobic–anaerobic microflora, which require the use of broad-spectrum antimicrobial agents as the most efficient treatment of clinically infected chronic wounds. Moreover, wound cleansing and surgical debridement may contribute to reducing microbial activity and infection, enhancing the selective action of antibiotics. In healthcare settings, infections often lead to prolonged hospitalization, the use of advanced dressings, and hence an increase in hospital costs [27]. Such healthcare predicament is currently compounded by the general aging of the global population and the growing incidence of diseases such as diabetes and obesity, which increase the social and economic impact of complicated wounds. The reduction of the bacterial load has been identified as crucial in wound management, and bacterial load represents one of the most important adverse factors. In view of the challenge posed by wound contamination, various wound dressings have been proposed to prevent chronicization and to support the healing process. Conventional wound dressings, including cotton and wool bandages, have been replaced by novel protective materials, which offer an adequate environment as well as provide elements to promote the healing process. It has been shown that hyaluronic acid (HA) activity is associated with multiple biological events during the wound healing process, promoting tissue repair and angiogenesis [28]. HA is a component of the extracellular matrix of the skin and decreases the infiltration of inflammatory cells, improves re-epithelization and granulation [29], and increases the formation of blood vessels. Moreover, HA is distinguished by its hydrophilicity, porosity, and swelling that support exudate absorption and cell migration and proliferation [30]. Roehrs and colleagues (2016) showed that HA could maintain a moist environment and thus assist cell migration to the wound bed [31]. The skin surrounding a wound is particularly vulnerable and, according to Hunter and colleagues (2013), the integrity of the periwound skin may be an important determinant in decreasing a wound size [32]. Several factors may contribute to periwound damage, such as exposure to the exudate and matrix metalloproteases (MMPs) [33]. HA has been shown to exert anti-inflammatory and anti-oedema effects [34]. The anti-inflammatory effects may be due to the action of exogenous HA as a scavenger through the draining of prostaglandins, MMPs, and other bioactive molecules. Therefore, HA allows the rapid diffusion of water-soluble molecules, enhances cell migration, and promotes a balanced wound environment. In addition, in animal models, HA was shown to be both a carrier for wound healing agents and an effective cosmetic product [35,36]. Any skin contamination affecting the periwound must be managed in a timely fashion to avoid the risk of potential wound infection. Wound bed colonization increases the risk of infection, with the likelihood of a greater risk of infection regarding the periwound area due to the wound exudate. Of the recent trends against burn infections involving the use of metal-based antimicrobials, the most prevalent one is silver [37]. Silver-containing mixtures have been the pillar of burn control, and silver sulfadiazine (SSD) has been the standard topical antimicrobial for complicated burns. SSD is adopted as a topical antibiotic for partial-thickness and full-thickness burns to counteract infection [38]. SSD is a broad-spectrum bactericidal antimicrobial that is effective against Gram-positive and Gram-negative bacteria, as well as some yeasts. SSD consists of silver and sulfadiazine. Silver ions released from SSD exert antibacterial effects through various mechanisms, i.e., membrane perturbation, DNA synthesis inhibition, and free radical production [39,40], while sulfadiazine, an antibiotic, may not be as effective. In our previous study [24], we hypothesized that this specific form of SSD may be essential to transport silver ions from sulfadiazine to the infected regions in wounds, highlighting the importance of silver ionization. A biofilm is a form of the bacterial life cycle and a settling structure on substances. Consequently, wound infections by bacteria are often associated with biofilms, exhibiting a 50-fold increased drug resistance compared to free bacteria on skin substrates [41]. In this regard, we analyzed bacterial killing concentrations vs. MIC using vancomycin and SSD. MBC/MIC and MBEC/MIC were 1/8 and 1/4 for SSD compared to vancomycin, showing that SSD was effective in destroying bacteria both in planktonic state and in biofilm. Furthermore, vancomycin administered via intravenous injection to patients may be ineffective in exerting an antibacterial action on the wound surface [24,41]. Moreover, we investigated the efficacy of SSD in mice models infected with *S. aureus*. The SSD treatment decreased bacterial colony proliferation in a dose-dependent manner on the wound but inhibited the development of tissue granulation at the highest dose (800 μg/wound). In light of these findings, uncertainty is expressed regarding SSD doses in a clinical context. Moreover, clinical data from this study indicate that the active combination of hyaluronic acid and silver sulfadiazine was able to reduce both the wound area and the bacterial load.

This retrospective observational study highlights the importance and effectiveness of adequately designed wound dressings, such as Connettivina Bio Plus^®^ cream and gauze pads. In addition, the presence of LMW Hyaluronic Acid promoted the granulating tissue, protected from bacterial biofilms, and improved the perilesional surrounding tissue. The improvement in ulcer healing was most likely due to the combined action of silver sulfadiazine and hyaluronic acid. HA, present in the formulation, creates a humid environment that encourages cell viability and regeneration and decreases fluid loss, supporting granulation tissue growth, which is directly proportional to wound closure. In association with HA, silver sulfadiazine, present in the formulation, provides a supplementary advantage to wound management, preventing bacteria growth and infection rate. In our study, bacterial management and wound bed management were strongly correlated to the improvement of periwound skin, a fundamental parameter in chronic ulcers, confirming that the presence of HA maintained an optimal moist environment and favored the healing process. Our results indeed showed a clear reduction in discomfort, erythema, and swelling. Based on these data, we used this dressing to cover wounds in the presence of inflammation and oedema but also to prevent microbial contamination.

This retrospective study presents limitations due to the lack of a control group as well as to the retrospective nature of the investigation. Therefore, we consider that the efficacy of the combination of hyaluronic acid and silver sulphadiazine requires further and independent evaluation for each of the different wound types. Albeit these limitations, the specific combined action of silver sulfadiazine and hyaluronic acid (in both cream and gauze formulations) yielded optimal outcomes of the healing process and aided in retaining a proper humid environment as well as in providing adequate protection of the wound from detrimental bacterial colonization.

## 5. Conclusions

In conclusion, this retrospective study demonstrates that the local application of hyaluronic acid and silver sulfadiazine, in both cream and gauze pad formulations, on wounds of mixed etiology showed significant statistic and clinical effectiveness in reducing wound size, protecting the surrounding skin, and preventing bacterial infection. In particular, the combination of LMW HA and SSD was effective from the first application in reducing the wound area and the bioburden. By promoting periwound health, HA was able to improve the physiological wound healing, decrease infection risk, reduce dressing frequency and its associated costs, reduce pain and discomfort, and improve quality of life. Furthermore, the treatment showed positive outcomes regarding patient tolerance, comfort, and overall contentment, as well as the reduction of inflammatory processes. Based on the results of this study and our experience, Connettivina Bio Plus^®^ cream and gauze pads may improve the management of chronic wounds and restrain infections and could be easily integrated with other therapies commonly applied in such clinical cases.

## Figures and Tables

**Table 1:** Patient characteristics at inclusion (*n* = 80).

Characteristic	Value
Age [mean ± SD]	67 ± 15
Gender [*n* (%)]	
Male	56 (70)
Female	24 (30)
Weight (Kg) [mean ± SD]	62.5 ± 14.5
Height (cm) [mean ± SD]	166 ± 4
Nutritional status [*n*]	
Good	62
Correct	16
Bad	2
Associated pathologies	
Respiratory system	6
Cardiovascular system	22
Cancer	10
Trauma (fractures)	60
Diabetes mellitus	10
Neurological disorders	5
Smoker	21
Ulcer Etiologies	
Diabetes	10
Post-traumatic ulcers	45
I- or II-degree Burns	15
Superficial abrasion	10
Duration [*n* (%)]	
<1 month	10 (12.5)
1–2 months	40 (50)
2–5 months	20 (25)
5–12 months	10 (12.5)
Location [*n* (%)]	
Heel	5 (6.25)
Sacrum	5 (6.25)
Tibia	10 (12.5)
Malleolus	25 (31.25)
Thigh	2 (2.5)
Finger	13 (16.25)
Hand	18 (22.5)
Forearm	2 (2.5)

**Table 2:** Baseline chronic ulcer characteristics for the study patients (*n* = 80).

Chronic Ulcer Characteristic	Value
Etiologies	
Diabetes	10
Post-traumatic ulcers	45
Burns	15
Superficial abrasion	10
Duration [*n* (%)]	
<1 month	10 (12.5)
1–2 months	40 (50)
2–5 months	20 (25)
5–12 months	10 (12.5)
Location [*n* (%)]	
Heel	5 (6.25)
Sacrum	5 (6.25)
Tibia	10 (12.5)
Malleolus	25 (31.25)
Thigh	2 (2.5)
Finger	13 (16.25)
Hand	18 (22.5)
Forearm	2 (2.5)
Granulation Tissue (%) [mean ± SD]	43 ± 8.6
Estimated surface area (cm^2^) [median (range)]	7.45 (3–45)
Estimated depth (cm) [median (range)]	0.35 (0–0.5)
Appearance of surrounding skin [*n* (%)]	
*Inflammation*	
Nil	0 (0)
Slight	29 (36.25)
Moderate	51 (63.75)
*Oedema*	
Nil	26 (32.5)
Slight	34 (42.5)
Moderate	20 (25)
*Purpura*	
Nil	0 (0)
Slight	15 (18.75)
Moderate	65 (81.25)
*Erythema*	
Nil	0 (0)
Slight	57 (71.25)
Moderate	23 (28.75)
Harikrishna Periwound Skin (HPS) classification [*n* (%)]	
Class 0	0 (0)
Class 1	0 (0)
Class 2a	0 (0)
Class 2b	0 (0)
Class 2c	0 (0)
Class 3	0 (0)
Class 4	80 (100)
Class 5	0 (0)
Pain (10 mm VAS) [mean ± SD]	4.5 ± 3.5

**Table 3:** The effect of vancomycin and SSD on a clinical isolate of *S. aureus*.

	State	MIC	MBC	MBEC	MBC/MIC	MBEC/MIC
Vancomycin (μg/mL)	Planktonic	1.56	1.56	NA	1	NA
	Biofilm	1.56	50	50	32	32
SSD (μg/mL)	Planktonic	125	250	NA	2	NA
	Biofilm	125	500	1000	4	8

Data are independent triplicated results. NA = not available.

## Data Availability

The clinical data used to support the findings of this study are included within the article.
